# Comparing the cut score for the borderline group method and borderline regression method with norm-referenced standard setting in an objective structured clinical examination in medical school in Korea

**DOI:** 10.3352/jeehp.2021.18.25

**Published:** 2021-09-27

**Authors:** Song Yi Park, Sang-Hwa Lee, Min-Jeong Kim, Ki-Hwan Ji, Ji Ho Ryu

**Affiliations:** 1 Department of Emergency Medicine, Dong-A University College of Medicine, Busan, Korea; 2 Department of Medical Education, Dong-A University College of Medicine, Busan, Korea; 3 Department of Medical Education and Neurology, Kosin University College of Medicine, Busan, Korea; 4 Department of Neurology, Inje University Busan Paik Hospital, Inje University College of Medicine, Busan, Korea; 5 Department of Emergency Medicine, Pusan National University School of Medicine, Busan, Korea; Hallym University, Korea

**Keywords:** Checklist, Educational measurement, Medical students, Objective structured clinical examination, Standard setting

## Abstract

**Purpose:**

Setting standards is critical in health professions. However, appropriate standard setting methods do not always apply to the set cut score in performance assessment. The aim of this study was to compare the cut score when the standard setting is changed from the norm-referenced method to the borderline group method (BGM) and borderline regression method (BRM) in an objective structured clinical examination (OSCE) in medical school.

**Methods:**

This was an explorative study to model the implementation of the BGM and BRM. A total of 107 fourth-year medical students attended the OSCE at 7 stations for encountering standardized patients (SPs) and at 1 station for performing skills on a manikin on July 15th, 2021. Thirty-two physician examiners evaluated the performance by completing a checklist and global rating scales.

**Results:**

The cut score of the norm-referenced method was lower than that of the BGM (P<0.01) and BRM (P<0.02). There was no significant difference in the cut score between the BGM and BRM (P=0.40). The station with the highest standard deviation and the highest proportion of the borderline group showed the largest cut score difference in standard setting methods.

**Conclusion:**

Prefixed cut scores by the norm-referenced method without considering station contents or examinee performance can vary due to station difficulty and content, affecting the appropriateness of standard setting decisions. If there is an adequate consensus on the criteria for the borderline group, standard setting with the BRM could be applied as a practical and defensible method to determine the cut score for OSCE.

## Introduction

### Background/rationale

Standard setting is a process by which human judgment can be synthesized in a rational and defensible way to classify score scales into categories [1]. Although there is no ‘gold standard’ in regard to setting the cut score in real examinations, this activity is critical in health professions [2]. This is the minimum judgment on the performance of medical practice to assess whether it is acceptable or unacceptable.

There are 2 types of standards: norm-referenced (relative) and criterion-referenced (absolute). For medical education examinations, a criterion-referenced standard is generally preferred. Because the norm-referenced standard tells little about the individual examinee, the cut score varies according to the competency level of the examinee group. Criterion-referenced standard setting methods are of 2 types: test-centered and examinee-centered. The former is appealing for setting a cut score on knowledge assessment, such as multiple-choice examination, and the latter is well suited for performance assessment, such as objective structured clinical examination (OSCE) [3]. Well-known examinee-centered standard settings are the contrasting groups method and the borderline group method (BGM).

In the BGM, examiners evaluate students’ performance on a global rating scale such as good, borderline, and fail. The cut scores are the median scores of examinees with borderline ratings. The BGM is time efficient and straightforward to implement. However, it has some limitations in that achieving consensus on the borderline group is difficult, and when the number of borderline examinees is small, the cut score may have low validity [4]. Recognizing the limitations of the BGM, the borderline regression method (BRM) was developed, which uses all OSCE checklist scores to develop a cut score using linear regression. Regression of global rating scores to OSCE total scores yields a linear equation. The predicted cut score of the borderline group is determined by substituting the borderline rating values into the regression equation.

However, it seems that examinee-centered standard-setting methods do not always apply to the setting of cut scores in performance assessments. In some instances, health care professional educators have used available assessment methods to assess a physician’s competencies even if the methods were not appropriate [5]. The cut score (usually 60 percentile) based on the norm-referenced standard setting is also used in the performance assessment [6]. This standard setting is easy to understand and apply. However, norm-referenced standard setting is difficult to justify because it does not consider the difficulty of the stations [4].

Inappropriate standard setting method can lead to undesirable result. There may be examinees who fail even though they are competent and examinees who pass even though they are not competent. This is an important issue for educational administrators as well as medical educators. However, there are few studies on standard setting and most of the studies have been about comparison of test-centered standard setting method such as Angoff, Ebel in medical and nursing educations in Korea [7-9]. Performance assessment is an important part of the medical profession. Therefore, examinee-centered standard setting method should also be considered as important, but there has been no research on this in Korea.

### Objectives

The research question of this study was how the cut scores changed when the standard setting method was changed from a norm-referenced method to the BGM and BRM in an OSCE for medical students. The aim of this study was to compare the cut score when the standard setting is changed from the norm-referenced method to the BGM and BRM in an OSCE of fourth-year medical students. The results of this study can be of practical help to educational administrators and medical educators who are in charge of the performance assessment.

## Methods

### Ethics statement

This study was approved by the Institutional Review Board of Dong-A University (IRB approval no., 2-1040709-AB-N-01-202106-HR-047-02). This study was not on human subjects or human-originated materials; thus, informed consent from subjects was not indicated.

### Study design

This was an explorative study to model the implementation of BGM and BRM for setting the standard by identifying and analyzing the cut score [10].

### Setting

This study was applied to the final day’s examination results of the Busan-Gyeongnam Clinical Skill Examination (BGCSE) conducted by the BGCSE consortium from July 12th to 15th, 2021. The consortium is an association of 5 medical schools in Busan-Gyeongnam region of South Korea that have annually conducted joint clinical skill examinations for the OSCE for third- and fourth-year medical students since 2014 [11].

The examination was comprised of 7 stations where students encountered standardized patients (SPs) and 1 station where students performed procedural skills on a manikin. The topic of each station was as follows: station 1, a 60-year-old woman presented with cough and shortness of breath for the past month; station 2, a 41-year-old woman presented with swelling and bruising of her right eye; station 3, a 44-year-old man with a right knee pain from a week ago; station 4, a 40-year-old woman with occasional vaginal bleeding for 2 months; station 5, a 26-year-old man with seizure this morning; station 6, a 46-year-old woman with sudden onset of dizziness after waking up this morning; station 7, a 21-year-old woman with right lower abdominal pain; and station 8, a 57-year-old man suddenly lost consciousness in a ward hallway. There were no newly added or developed stations for this study.

The examiners’ training proceeded in the same way as usual. A total of 32 physician examiners evaluated examinee performance at each station in 4 medical schools by completing the checklist and global rating scales. The only change was that the existing 4-point numeric scale for proficiency in global rating was changed to a categorical scale of fail, borderline, good, and excellent. The cut score of each station was determined as follows: (1) calculate the mean and standard deviation (SD); (2) subtract 1 SD from the mean; and (3) set this score as the cut score.

### Participants

A total of 107 fourth-year medical students from 5 medical schools attended the last day of the BGCSE at 4 medical school skill centers.

### Variables

The primary outcomes were defined as a cut score by the norm-referenced method, BGM, and BRM of each station. The cut score of norm-referenced method was determined by subtracting 1 SD from the mean of each station, which is the conventional method in BGCSE. The cut score of the BGM was performed by the following steps: (1) borderline group examinees were identified, (2) their checklist scores were collected, and (3) the median score for this group was set as the cut score. The cut score of the BRM was determined as following steps: (1) checklist and global rating scores of all examinees at the station were collected, (2) a regression equation (y=a+bx) was produced using Microsoft Excel (Microsoft Corp., Redmond, WA, USA), (3) the scale of borderline group (in this study, x=2) was inserted into the equation, and (4) the calculated y of the equation was set as the cut score.

The secondary outcome was defined as the number of failed students at each station according to each standard setting method.

### Data sources/measurement

The examiners scored the students’ performance using a computer program, and the results were automatically processed. All variables were recorded in an Excel spreadsheet (Microsoft Corp.).

### Bias

No bias was found in the study scheme.

### Study size

This study was not intended to determine effect and was therefore not indicated to calculate sample size.

### Statistical methods

Descriptive statistics were used, including the mean and SD of each station and borderline group. Regression analysis was conducted to produce a regression equation using Microsoft Excel ver. 2105 (Microsoft Corp.). The scale of the borderline group (x=2) was inserted into the regression equation to calculate the cut score of the BRM. A paired t-test for the cut score comparison between the norm-referenced method and BGM, and between the norm-referenced method and BRM were conducted. The P-value <0.05 was considered significant.

## Results

### Participants

A total of 107 students completed the examination, and 32 professors participated as examiners.

### Main results

The reliability using the G-coefficient in 7 SP encounter stations and 1 skill station was 0.76 and 0.73, respectively (Tables 1, 2).

#### Predicted cut score of each station by the norm-referenced standard setting method, BGM, and BRM

**Fig. 1.**
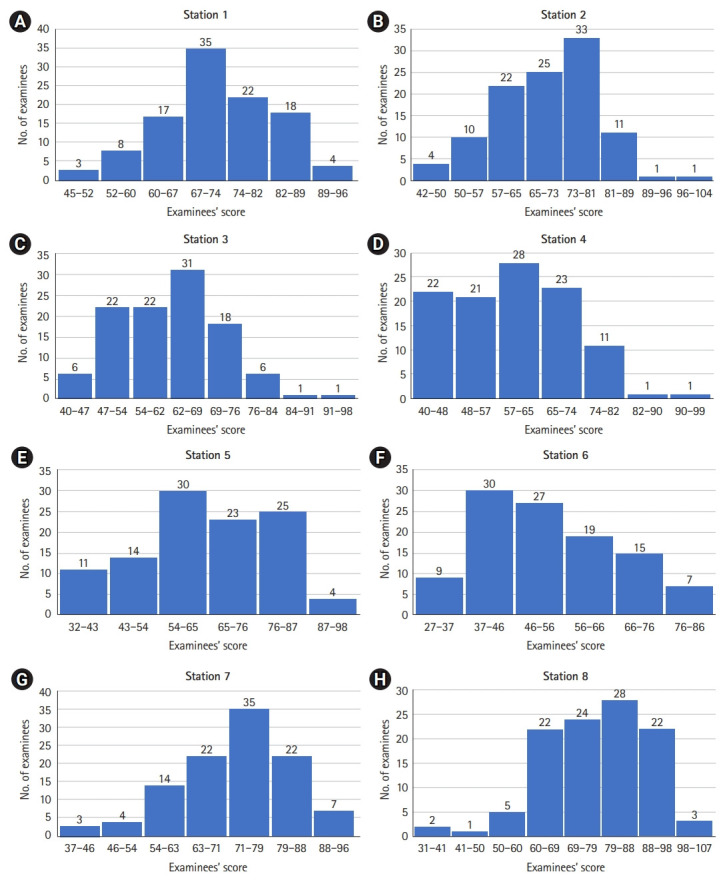
(A–H) The histogram of each station in objective structured clinical examination (OSCE). The x-axis is the examinees’ scores, and the y-axis is the number of examinees. A total of 107 examinees participated in each station, and the number indicated on the bar is the number of examinees in the corresponding score section.

**Fig. 2.**
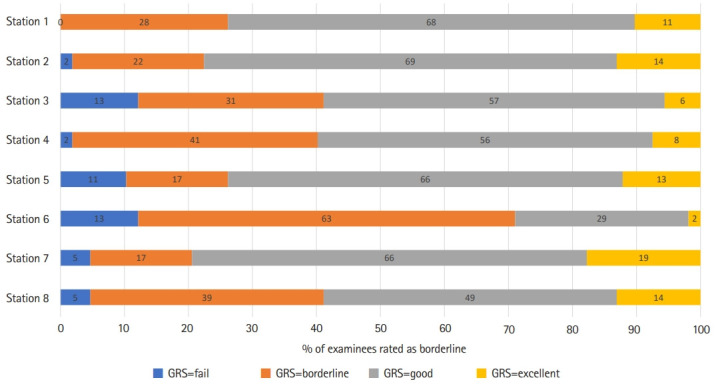
The proportion of examinees rated as the borderline group of each station in the objective structured clinical examination. The number presented on the on colored bar is the percentage of examinees rated as borderline. The x-axis is the percentage of rated as borderline and y-axis is station number. GRS, global rating scale.

**Fig. 3.**
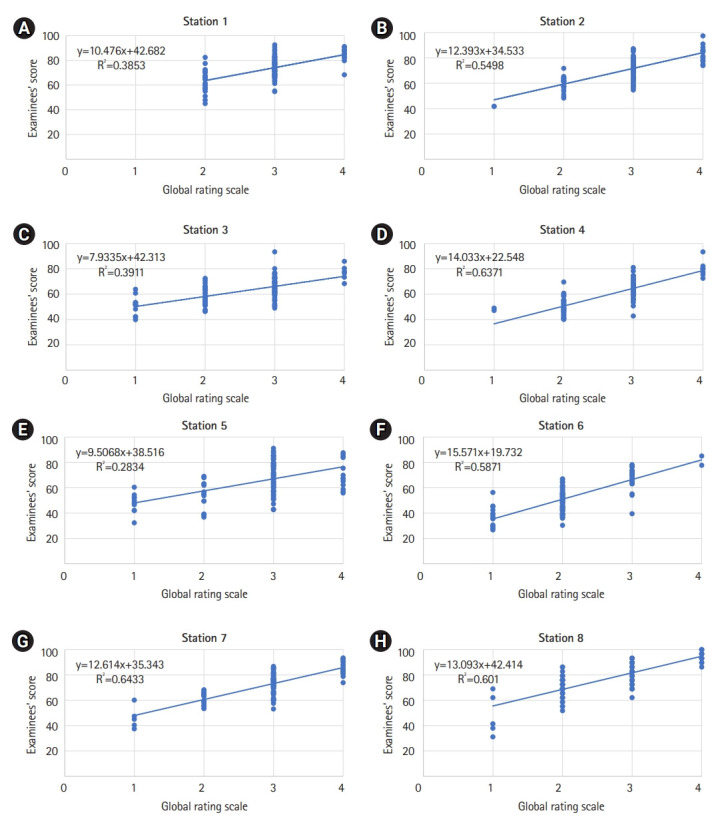
(A–H) The regression plot of each station in the objective structured clinical examination. The x-axis is the global rating scale, and the y-axis is the examinees’ score at each station. The regression equation and the correlation of determination (R^2^) are presented.

The mean, SD, and predicted cut score by the norm-referenced method and the BGM and BRM of each station are shown in Table 3 and Dataset 1. The histogram of the examinees’ scores is shown in Fig. 1. The proportion of examinees rated as “borderline” at each station is shown in Fig. 2. The regression equation, correlation of determination (R^2^), and plot of each station by the BRM are shown in Table 4, Dataset 1, and Fig. 3.

The cut score of the norm-referenced method was lower than that of the BGM in each station (P<0.01) and BRM (P<0.02), respectively. There was no significant difference in the cut score between the BGM and BRM (P=0.99) (Table 3). Station 5, with the highest SD, and station 6, with the highest proportion of examinees rated as “borderline”, showed the largest cut score difference by standard setting methods (Table 3). The correlation of determination (R^2^) of each station ranged from 0.28 to 0.64 (Table 4).

#### Number of examinees below standard by norm-referenced standard setting, BGM, and BRM

At all stations, there were more failed examinees by the BGM and BRM than by the norm-referenced standard setting method. In particular, the number of failed examinees at stations 3, 5, and 6 almost doubled (Table 1).

## Discussion

### Key results

This study aimed to compare the cut score when the standard setting was changed from the norm-referenced method to the BGM and BRM in an OSCE of fourth-year medical students. The overall cut scores of the BGM and BRM were similar or higher than those of the norm-referenced method; thus, the number of failed examinees was higher. However, stations 3, 5, and 6 showed the largest differences in the cut score according to each standard setting method.

### Interpretation

The standard setting for OSCEs is important; however, there are few practical guidelines that handle real medical students’ data and compare the outcomes of using different standard setting methods. Comparing the characteristics of these standard setting methods would be useful for examination administrators of medical schools. This study reported and provided a real example of the implementation of 2 standard setting methods for OSCEs.

Stations 3 and 6 seemed to have a high level of case difficulty considering their histograms (Fig. 1). If the station is difficult, there may be many examinees with low scores. Under the BGM and BRM, which are based on actual examinee performance, failed examinees would increase. However, the norm-referenced method (usually set below 1 SD or 60 percentile) does not consider the difficulty of the station; it simply defines failed examinations as below 1 SD. The difference in cut scores according to standard settings at stations 3 and 6 shows that the norm-referenced method does not function well in difficult stations.

Station 5 had the lowest proportion of examinees rated as borderline (15.89%) and the highest SD (14.27). If there are insufficient cohorts evaluated as borderline, cut scores may be calculated based on a relatively small number of examinees, which may increase the statistical error associated with the cut score [12]. As the score distribution is left-skewed and the borderline group is at the lower thin tail of the overall score distribution, the mean or median will be biased toward the high side [13]. This bias can also be confirmed in the results of this study. In station 6 with the largest borderline group, the difference in cut score between BGM and BRM was 0.20 (the smallest difference among stations), but at station 5 with the smallest borderline group, it was 2.86 (the largest difference among stations). At station 5, the difference in cut scores between the BGM and BRM is thought to be due to this bias. If the cohort of borderline group is smaller, this difference will be larger. However, linear regression uses all the scores within the group and therefore avoids this bias.

### Comparison with previous studies

As the results of this study show, the norm-referenced method had a risk of examinees passing the station even though the examiner evaluated the examinee as not competent on the performance. This type of standard setting is typically used when selecting applicants for employment or for educational programs where available positions are limited [14]. The setting of standards should be applied according to the goal of the examination. If the OSCE is not used for applicant selection in medical school, the norm-referenced method is not appropriate.

The advantages of the BGM are that no statistical procedure is required and the cut score calculation is easy. However, the BGM can have a potential problem when the borderline group is not sufficient, such as in station 5 of this study. The same problem was also found in the study of Wood et al. [13]. In their study, the borderline group was 20% (12/59 examinees), and the difference in the pass rate was 69% in the BGM and 92% in the BRM. When the station was reviewed in detail, all 12 examinees had borderline satisfactory scale (they used 6-point scales with inferior, poor, borderline unsatisfactory, borderline satisfactory, good, and excellent), indicating that the cut score of the BRM was a more appropriate reflection of the examinees’ performance.

Most studies using the BRM have been conducted in the evaluation of relatively large cohorts (n>50) in which the examinee group is high performing, such as postgraduate candidates. This study was also conducted in a large cohort of 107 examinees. However, OSCEs in many medical schools may have small cohorts, such as a single-year group. Homer et al. [12] have shown that the use of the BRM in the context of small cohorts can be generally successful. They investigated the use of the BRM in different high stakes assessment contexts and found that the BRM functions effectively at most stations. They proposed an extant cut score from a practical point of view. Extant cut scores based on previous station performance would ideally be available in a small cohort [12].

In the BRM, to assess the fitness of the regression model in terms of how well the model predicts the cut score of the OSCE, the R^2^ is examined. R^2^ is generally interpreted as a percentage of the score achieved in an examination that can be explained by a benchmark score of global rating on examinees’ clinical performance. An R^2^ of 1.0 means that all scores of the checklist are completely explained by the global rating scale of the examiner as an independent variable. A high R^2^, between 0.85 and 1.0, indicates that the checklist of examinees’ clinical performance is aligned with the examiner’s evaluation reflected in the global rating score. A low R^2^ of 0.5 or less indicates that the checklist score is not aligned with the global rating score [15]. However, in many studies, the value of R^2^ was approximately 0.5 and was considered reasonable, which was not significantly different from this study [16,17].

### Limitations

The consensus for rating borderline groups of students by examiners is important for the BGM and BRM. However, this study did not address this point. We assumed that the examiners who participated in the BGCSE had sufficient consensus based on their many years of scoring experience. In order to maintain this consensus, the 4 categorical scale, which examiners have been accustomed to using for many years, was used without modification as global rating scale. However, this assumption will not always be valid. If the medical school does not have enough experienced examiners, it may be helpful to develop a model to identify borderline groups of students [18].

### Generalizability

Considering the results of this study, the BRM can be applied to a small cohort, and its statistical methods are at a level that can be performed using Microsoft Excel (Microsoft Corp.).

### Suggestions

The global rating scale used in this study was a categorical scale including fail, borderline, good, and excellent. However, further study is needed to determine how the cut score changes when this scale changes to 5 or 6. In the OSCE of this study, there were 7 SP encounter stations and 1 skill station. It is also necessary to study how the cut score changes depending on the content and combination of the stations.

### Conclusions

The cut score of the norm-referenced method was lower than that of the BGM and BRM, and there was no significant difference in the cut score between the BGM and BRM. It will not be easy to change the previously used standard setting method. However, prefixed cut scores by the norm-referenced method, without considering station contents or examinee performance, can vary due to station difficulty and content, affecting the appropriateness of the standard setting decision. If the cut score of OSCE is the minimum judgement assessing whether the performance of medical practice is acceptable or not, examinee-centered standard setting method are more appropriate for that purpose. Moreover, as presented in this study, BGM and BRM methods are not difficult to apply in practice. If there is an adequate consensus on the borderline group criteria, standard setting with the BRM could be applied as more defensible method to determine the cut score of the OSCE stations.

## Figures and Tables

**Table 1.** G-study results for the checklist score for 7 standardized patient encounter stations in an objective structured clinical examination of fourth-year medical students

Effect	df	T-value	SS	MS	VC	% of VC
p	106	278.61614	278.61613	2.62845	0.012	2.22
c	6	137.15853	137.15852	22.85975	-0.00229	0.41
i:c	154	4469.28246	4332.12393	28.13067	0.26042	46.75
pc	636	820.26218	404.48752	0.63599	0.01610	2.89
pi:c	16,324	9492.77797	4340.39185	0.26589	0.26589	47.73

The variables of the effect are as follows: person (p), case (c), and item (i). The model of p*(i:c) were used in G-string ver. 6.3.8 (Ralph Bloch, Hamilton, ON, Canada). Phi-coefficient=0.689. G-coefficient=0.758. df, degrees of freedom; SS, sum of squares; MS, mean square; VC, variance components.

**Table 2.** G-study results for the checklist score for 1 procedural skill station in an objective structured clinical examination of fourth-year medical students

Effect	df	T-value	SS	MS	VC	% of VC
p	106	52.73098	52.73097	0.49746	0.01289	7.32
I	27	80.73565	80.73565	2.99021	0.02667	15.14
pi	2,862	524.23098	390.76435	0.13654	0.13654	77.54

The variables of the effect are as follows: person (p), case (c), and item (i). The model of p*i was used in G-string ver. 6.3.8 (Ralph Bloch, Hamilton, ON, Canada). Phi-coefficient=0.689. G-coefficient=0.726. df, degrees of freedom; SS, sum of squares; MS, mean square.

**Table 3.** Predicted cut score by norm-referenced, borderline group and borderline regression method in OSCE of fourth-year medical students

Variable	Station 1	Station 2	Station 3	Station 4	Station 5	Station 6	Station 7	Station 8	P-value
OSCE score									
Maximum	92.48	97.33	93.33	93.33	91.11	85.06	93.33	92.48	
Minimum	45.03	41.75	39.89	40	32.22	26.67	37.44	45.03	
Mean	72.45	70.32	62.33	59.8	64.73	53.78	72.24	72.45	
SD	9.88	10.6	9.91	11.35	14.27	13.43	11.37	9.88	
Number rated as borderline	28	22	31	41	17	63	17	39	
% rated as borderline	26.17	20.56	28.97	38.32	15.89	58.88	15.89	36.45	
Norm-referenced method (below one SD from mean)									
Predicted cut score	62.57	59.72	52.42	48.44	50.46	40.36	60.87	64.54	
Number below standard	15	17	19	22	18	21	19	13	
% below standard	14.02	15.89	17.76	20.56	16.82	19.63	17.76	12.15	
Borderline group method									
Predicted cut score	65.64	61.5	56.08	48.49	54.67	51.07	63	68.97	<0.01^a)^
Number below standard	20	20	33	22	29	48	21	30	
% below standard	18.69	18.69	30.84	20.56	27.1	44.86	19.63	28.04	
Borderline regression method									
Predicted cut score	63.63	59.32	58.18	50.61	57.53	50.87	60.57	68.6	<0.02^a)^
Number below standard	17	17	37	27	36	47	18	16	0.99^b)^
% below standard	14.85	14.85	32.32	22.71	20.09	38.43	17.47	12.15	

OSCE stations 1–7 were interview-based examination and OSCE station 8 was skill-based examination. The P-value of <0.05 was considered significant. OSCE, objective structured clinical examination, SD, standard deviation.

a) The cut score of norm-referenced methods was lower than that of borderline group method (P<0.01) and borderline regression method (P<0.02) by paired t-test.

b) There was no significant difference in cut score between borderline group method and borderline regression method (P=0.99) by paired t-test.

**Table 4.** Regression equation of borderline group method of each station

Borderline regression method	Station 1	Station 2	Station 3	Station 4	Station 5	Station 6	Station 7	Station 8
Regression equation	y=10.476x+42.682	y=12.393x+34.533	y=7.9335x+42.313	y=14.033x+22.548	y=9.5068x+38.516	y=15.571x+19.732	y=12.614x+35.343	y=13.093x+42.414
Significance F	0.00	0.00	0.00	0.00	0.00	0.00	0.00	0.00
P-value	0.01	0.01	0.01	0.01	0.01	0.01	0.01	0.01
R^2^	0.39	0.55	0.39	0.64	0.28	0.59	0.64	0.60

A P-value of <0.05 was considered significant.

## Footnotes

**Data availability**
Data files are available from Harvard Dataverse: https://doi.org/10.7910/DVN/CY7XUN Dataset 1. Raw score data of examinees for each station.

## Supplementary materials

Supplement 1. Audio recording of the abstract.

## References

[1] De Champlain AF, Swanwick T, Forrest K, O'Brien BC (2018). Understanding medical education: evidence, theory, and practice.

[2] Kane MT, Crooks TJ, Cohen AS (1999). Designing and evaluating standard-setting procedures for licensure and certification tests. Adv Health Sci Educ Theory Pract.

[3] Kaufman DM, Mann KV, Muijtjens AM, van der Vleuten CP (2000). A comparison of standard-setting procedures for an OSCE in undergraduate medical education. Acad Med.

[4] Reid KJ, Dodds A (2014). Comparing the borderline group and borderline regression approaches to setting objective structured clinical examination cut scores. J Contemp Med Educ.

[5] Norcini JJ, McKinley DW (2007). Assessment methods in medical education. Teach Teach Educ.

[6] Liu M, Liu KM (2008). Setting pass scores for clinical skills assessment. Kaohsiung J Med Sci.

[7] Park J, Yim MK, Kim NJ, Ahn DS, Kim YM (2020). Similarity of the cut score in test sets with different item amounts using the modified Angoff, modified Ebel, and Hofstee standard-setting methods for the Korean Medical Licensing Examination. J Educ Eval Health Prof.

[8] Park J, Ahn DS, Yim MK, Lee J (2018). Comparison of standard-setting methods for the Korea Radiological Technologist Licensing Examination: Angoff, Ebel, Bookmark, and Hofstee. J Educ Eval Health Prof.

[9] Yim MK, Shin S (2020). Using the Angoff method to set a standard on mock exams for the Korean Nursing Licensing Examination. J Educ Eval Health Prof.

[10] Ringsted C, Hodges B, Scherpbier A (2011). “The research compass”: an introduction to research in medical education: AMEE guide no. 56. Med Teach.

[11] Park SY, Kong HH, Kim MJ, Yoon YS, Lee SH, Im S, Seo JH (2020). Clinical performance of medical students in Korea in a whole-task emergency station in the objective structured clinical examination with a standardized patient complaining of palpitations. J Educ Eval Health Prof.

[12] Homer M, Fuller R, Hallam J, Pell G (2020). Setting defensible standards in small cohort OSCEs: understanding better when borderline regression can ‘work’. Med Teach.

[13] Wood TJ, Humphrey-Murto SM, Norman GR (2006). Standard setting in a small scale OSCE: a comparison of the Modified Borderline-Group Method and the Borderline Regression Method. Adv Health Sci Educ Theory Pract.

[14] McKinley DW, Norcini JJ (2014). How to set standards on performance-based examinations: AMEE guide no. 85. Med Teach.

[15] Homer M, Pell G (2009). The impact of the inclusion of simulated patient ratings on the reliability of OSCE assessments under the borderline regression method. Med Teach.

[16] Yazbeck Karam V, Park YS, Tekian A, Youssef N (2018). Evaluating the validity evidence of an OSCE: results from a new medical school. BMC Med Educ.

[17] Hejri SM, Jalili M, Muijtjens AM, Van Der Vleuten CP (2013). Assessing the reliability of the borderline regression method as a standard setting procedure for objective structured clinical examination. J Res Med Sci.

[18] Ra’oof RA, Elaraby S, Abdelgawad E (2020). Developing a model for identification of the borderline group in objective structured clinical exam. Educ Med J.

